# Reference ranges for serum insulin-like growth factor I (IGF-I) in healthy Chinese adults

**DOI:** 10.1371/journal.pone.0185561

**Published:** 2017-10-04

**Authors:** Huijuan Zhu, Yuan Xu, Fengying Gong, Guangliang Shan, Hongbo Yang, Ke Xu, Dianxi Zhang, Xinqi Cheng, Zhihao Zhang, Shi Chen, Linjie Wang, Hui Pan

**Affiliations:** 1 Department of Endocrinology, Key Laboratory of Endocrinology of National Health and Family Planning Commission, Peking Union Medical College Hospital, Chinese Academy of Medical Science & Peking Union Medical College, Beijing, China; 2 Department of Surgery, Peking Union Medical College Hospital, Chinese Academy of Medical Science & Peking Union Medical College, Beijing, China; 3 Department of Epidemiology and Statistics, Institute of Basic Medical Sciences, Peking Union Medical College Hospital, Chinese Academy of Medical Science & Peking Union Medical College, Beijing, China; 4 Department of Laboratory Medicine, Peking Union Medical College Hospital, Chinese Academy of Medical Science & Peking Union Medical College, Beijing, China; 5 Haas School of Business, University of California, Berkeley, California, United States of America; Department of Neurology, University of California, San Francisco, California, United States of America; Neurocenter of Southern Switzerland, SWITZERLAND

## Abstract

**Purpose:**

To determine serum insulin-like growth factor 1 (IGF-I) levels in healthy Chinese adults, establish reference ranges for serum IGF-I levels and observe the effects of age, sex, body mass index (BMI) and geographical region on serum IGF-I levels.

**Methods:**

In total, 2791 healthy adults (1339 males and 1452 females) from the north (Beijing) and south (Guizhou Province) of China were recruited following a questionnaire survey, physical examination and laboratory examination. Both sexes were divided into 13 groups according to age (18, 19, 20–24, 25–29, 30–34, 35–39, 40–44, 45–49, 50–54, 55–59, 60–64, 65–69 and ≥70 years). The serum IGF-I levels were measured by performing a chemiluminescent assay (Immulite 2000®), and the LMS (Lambda-Mu-Sigma) method was applied to construct smooth centile curves of age-specific IGF-I levels.

**Results:**

**S**erum IGF-I levels in the adults gradually decreased with increasing age from 18 to 70 years in both the male and female participants. Although the decrease in the level of IGF-1 was more pronounced in females than in males, no significant difference was observed between the sexes, except in the 60- to 64-year-old age group (P = 0.0329). The multiple linear regression model showed that there was an inverse relationship between the serum IGF-I level and BMI (P<0.001), and the serum IGF-I level in the Guizhou population was higher than that in the Beijing population (P<0.05).

**Conclusion:**

The normal reference ranges for age- and sex-specific serum IGF-I levels were established for the first time in a large sample of Chinese adults. The serum IGF-I levels were significantly influenced by age, BMI and geographical region.

## Introduction

Insulin-like growth factor I (IGF-I) regulates cell proliferation. Human IGF-I was first discovered by Salmno and Daughaday in 1975 [1], and its chemical structure was identified in 1978 [2]. Over 70% of the IGF-I found throughout the body is synthesized in the liver under the regulation of growth hormone (GH); thus, the serum IGF-I levels could reflect endogenous GH secretion [3]. Compared with GH, which has a pulsatile release pattern, IGF-I has a much more stable pattern of release, and its half-life is approximately 18 to 20 hours.

Measurements of serum IGF-I are particularly useful in the diagnosis and management of patients with GH-IGF-I axis-related disorders, such as growth hormone deficiency (GHD) and excess GH secretion in children and adults. Moreover, serum IGF-I levels are used in dose adjustment for GH replacement therapy and have been considered the most reliable component for monitoring the effect of therapeutic interventions in acromegaly [1,4].

Juul et al. reported the reference ranges for adult serum IGF-I in Denmark in 1994 [5]. Subsequently, several related studies conducted in European and South American adults established the normal reference ranges for serum IGF-I in terms of age and sex [6]. The only Asian study was conducted in a Japanese population in 2012 [3] and measured the serum IGF-1 levels in 1685 healthy Japanese subjects from 0 to 83 years of age using immunoradiometric assays, which are not widely used. In addition to age, sex and physiological condition, the serum IGF-I levels also vary with ethnicity [7,8]. Liao et al. recently published reference IGF-I levels in a population from South China [9]. However, the number of adults between 18–20 years of age and the number of enrolled subjects were limited. Therefore, it is necessary to establish normal reference ranges for serum IGF-I in the Chinese population on a large scale.

Therefore, in our present study, we recruited a total of 2791 Han subjects from North and South China and measured their serum IGF-I levels using a chemiluminescent assay, which is widely used in both China and globally. Finally, this is the first study to establish the normal reference ranges for age- and sex-specific serum IGF-I levels in a large sample of Chinese adults. We found that the serum IGF-I levels gradually decreased with age in both sexes and that the serum IGF-1 levels correlated with the body mass index (BMI) and geographical region.

## Materials and methods

### Study population

This population-based study was conducted in North (Beijing City) and South (Guizhou Province) China between October 2009 and March 2012. A questionnaire survey, physical examination and laboratory measurements were performed to collect general information (sex, birthday, age, etc.), medical history (liver diseases, renal diseases, cancer, diabetes mellitus, diseases of the pituitary gland, pregnancy, drug history, etc.), physical parameters (height, weight, waistline, blood pressure, body fat percentage, fat mass, fat-free mass, etc.), urine parameters (urine glucose, urine protein, urine red blood cells, etc.), blood parameters (white blood cell count, hemoglobin, platelet count, etc.) (Siemens Advia 2120) and blood biochemical parameters (glucose, alanine aminotransferase, aspartate aminotransferase, γ-glutamyltransferase, total cholesterol, triglycerides, high-density lipoprotein, low-density lipoprotein, blood urea nitrogen, uric acid, creatinine, etc.) (OlympusAU5800). Information regarding the use of oral contraceptives was not collected.

The exclusion criteria were as follow: (1) subjects with liver diseases, renal diseases, cancer, diabetes mellitus, diseases of the pituitary gland and pregnancy (exclusion criteria included alanine aminotransferase > +2 SD, aspartate aminotransferase > +2 SD, γ-glutamyltransferase > +2 SD, platelet count > +2 SD, eGFR < 90 mL/min·1.73 m^2^, urine protein positive, any treatment for diabetes mellitus, pituitary gland hormone replacement therapy, and medical history); (2) subjects using potentially interfering medications, such as estrogen and corticosteroids; and (3) subjects with a BMI ≤18.5 kg/m^2^ or >28 kg/m^2^ who were defined as too thin or obese in the Chinese population, respectively.

In total, 2791 Han Chinese subjects (1339 males and 1452 females) were enrolled in the study. All subjects were divided into 13 groups according to age (18, 19, 20–24, 25–29, 30–34, 35–39, 40–44, 45–49, 50–54, 55–59, 60–64, 65–69 and ≥70 years). The number of participants in each group is shown in S1 Table. Each group consisted of more than 30 subjects, except for the age 19 group (9 males, 15 females, 24 in total).

This study was approved by the Ethics Committee of Peking Union Medical College Hospital (HS-1050). All participants provided written informed consent before participating in the study.

### Measures

All blood samples were drawn from the cubital vein between 8:00 a.m. and 9:00 a.m. after an overnight fast. The samples were collected in a BD vacutainer without any anticoagulation. The samples were then centrifuged at 3000 rpm for 15 min at 4°C, and the serum was transferred into Eppendorf tubes (2 ml). All samples were immediately frozen at -80°C until the measurements were performed. All samples were de-labeled; thus, individual participants could not be identified.

The serum IGF-I level was measured using a fully automated two-site, solid-phase, chemiluminescent enzyme immunometric assay (Immulite 2000®, Siemens Healthcare Diagnostics, Incorporated Gwynedd, UK) between April 12^th^, 2016, and June 7^th^, 2016. The assay was calibrated against the WHO NIBSC 1st IRR 87/518 criteria before performing the measurements. The Immulite IGF-I control module provided by the manufacturer was used as the inter-assay control, which included IGF-I standard samples with low (62.3 ng/ml), middle (270.0 ng/ml) and high (575.0 ng/ml) concentrations. All inter-assay controls were within ±2 SD values as defined by the manufacturer (details are shown in S2 Table). To better assess the accuracy of each measurement, another type of intra- and inter-assay control was used. We pooled human serum from medical examinations with unknown IGF-I concentrations and then thoroughly mixed and dispensed the serum into 47 Eppendorf tubes. Then, the controls were evenly added to the samples and measured in each measurement. The mean value was 216.7 ng/ml, and the total coefficient of variation (CV) was 3.04%. Detailed information is provided in S1 Fig.

### Statistical analyses

The construction of the centile curves was performed using the LMS method, which fits smooth centile curves to the reference data of age-specific serum IGF-I [10]. This method summarizes the percentiles at each age group based on the power of the age-specific Box-Cox power transformations that were used to normalize the data. The final percentile curves were produced according to three curves representing skewness (L curve), the median (M curve) and the coefficient of variation (S curve). The L, M, and S values were smoothed for each age and sex using cubic spline curves. For an individual IGF-I measurement (X), the standard deviation score (SDS) was calculated using the age-specific L, M, and S parameters and Eq 1 as follows:

$$SDS=\left[{\left(X/M\right)}^{L}\text{-}1\right]/\left(L\times S\right).$$ Eq 1

Then, the centile could be calculated using Eq 2 as follows:

$$IGF\text{-}I\;centile=M\times {\left(1+L\times S\times SDS\right)}^{1/L}$$ Eq 2

The fit of the centile curves was assessed by a visual inspection comparing the empirical centiles with the LMS-generated curves. The goodness of fit test was conducted to compare the simulated centiles and measured centiles.

The t-test and Wilcoxon signed ranks test were applied to analyze the influence of sex on the serum IGF-I levels and compare the difference between our results and those obtained by Elmlinger. A multiple linear regression model was also built to observe the influence of age, sex, BMI, geographical region, etc. on the serum IGF-1 levels. SAS (Statistical Analysis System, version 9.2, SAS Institute, Cary, N.C., USA)/Insight module [11] and IBM SPSS Statistics (version 21.0, IBM Co., USA) were used. Statistical significance was set at P<0.05.

## Results

### Reference ranges for serum IGF-I levels

The characteristics of all subjects are shown in S3 Table. The mean age and BMI were 44.9±14.8 years and 22.9±2.5 kg/m^2^, respectively. Age, BMI, TC, TG, and LDL in the males were all significantly higher than those in the females (P<0.01), while LDL and the fat % in the females were significantly higher than those in the males (P<0.01).

**Fig 1:**
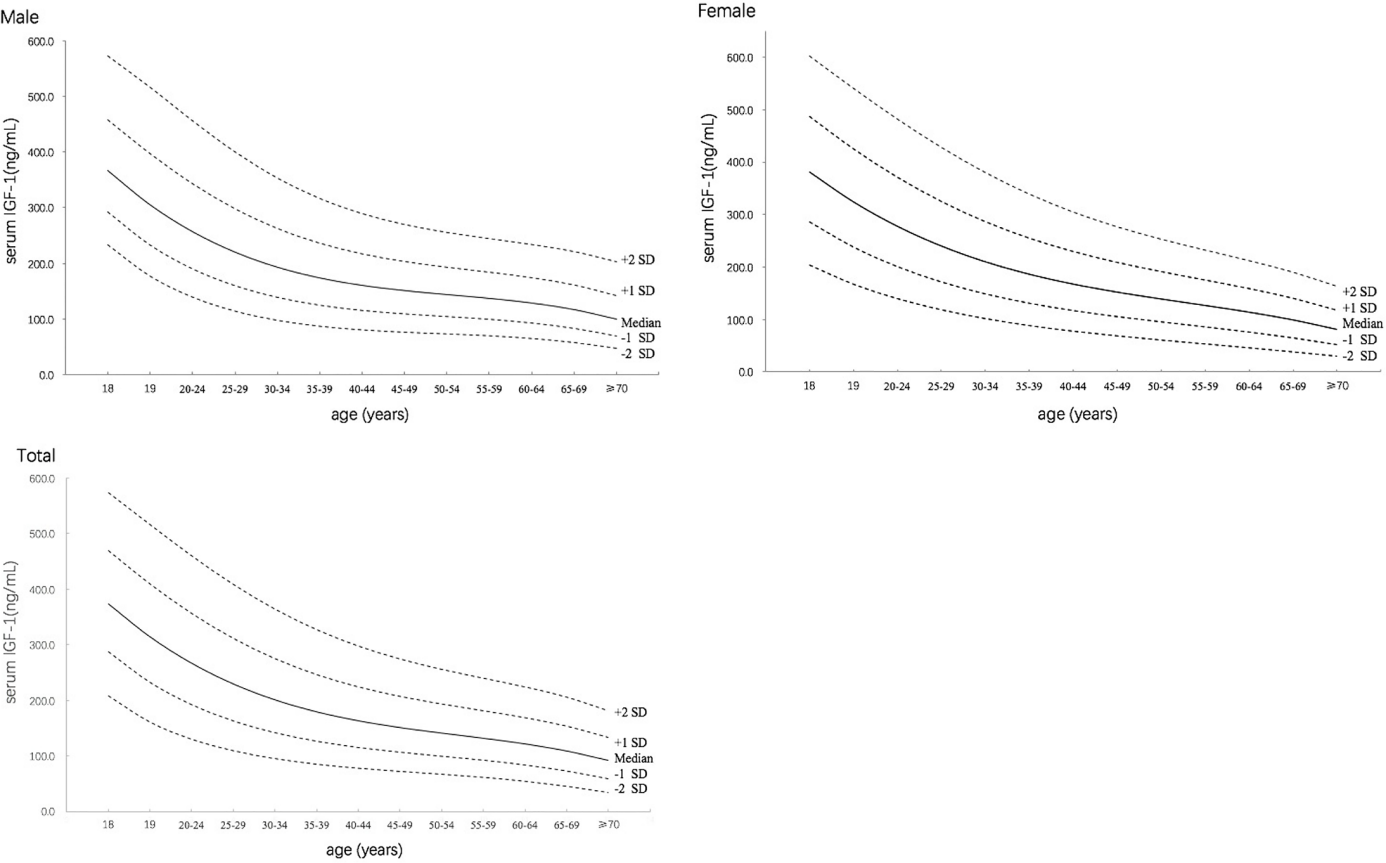
Curves showing +2SD, +SD, median, -SD, and -2SD of serum IGF-I levels in healthy Chinese adults (males, females and total).

The changes in the curves of the serum IGF-I levels with increasing age were drawn for the males, females and both sexes using the LMS method. As shown in Fig 1, the serum IGF-I levels gradually decreased with increasing age from 18 to 70 years in both sexes. The median serum IGF-I level was 374.1 ng/ml at the age of 18. The serum IGF-I level decreased to 180.1 ng/ml at the age of 35–39, which is 48.1% of that at age 18, and further decreased to 92.7 ng/ml at ages older than 70, which is approximately 24.8% of that at age 18. This trend was similar in both the females and males, although the degree of reduction was steeper in the females. For the convenience of clinical practice, the calculated median, ±2 SD, and ±1 SD of the serum IGF-I levels; the L, M, and S values; and the 2.5 and 97.5 percentile values are provided in tables (Table 1 for males, Table 2 for females, and Table 3 for the total). Each table also shows the L, M, and S values required to calculate the SDS using Eq 1.

**Table 1:** Serum IGF-I reference ranges and L, M, and S parameters of IGF-I in healthy male Chinese adults.

Age Group	L	M	S	Standard Deviation					Percentile	
				-2 SD	-1 SD	M	+1 SD	+2 SD	P_2.5_	P_97.5_
18	0.010	366.2	0.225	233.4	292.4	366.2	458.4	573.4	235.5	568.3
19	0.055	304.7	0.268	176.8	232.6	304.7	397.6	516.8	178.8	511.5
20–24	0.102	256.5	0.297	139.0	189.8	256.5	343.7	456.5	140.8	451.4
25–29	0.148	219.9	0.314	113.8	159.5	219.9	298.7	400.5	115.4	395.9
30–34	0.190	192.8	0.321	97.2	138.4	192.8	263.3	353.4	98.6	349.3
35–39	0.226	173.5	0.322	86.5	124.2	173.5	236.7	316.5	87.9	312.9
40–44	0.253	160.1	0.319	79.9	114.9	160.1	217.6	289.1	81.1	286.0
45–49	0.269	150.8	0.314	75.8	108.6	150.8	203.9	269.5	76.9	266.6
50–54	0.271	143.6	0.311	72.7	103.8	143.6	193.6	255.3	73.8	252.6
55–59	0.256	136.8	0.312	69.3	98.8	136.8	184.7	244.1	70.4	241.4
60–64	0.221	128.4	0.320	64.5	92.2	128.4	174.9	233.4	65.4	230.8
65–69	0.165	116.6	0.336	57.1	82.5	116.6	161.8	220.7	58.0	218.1
≥70	0.084	99.5	0.365	46.8	68.7	99.5	142.7	202.3	47.6	199.5

L, coefficient of skewness; M, Median; S, coefficient of variation. Both the standard deviation and percentile values are shown here for clinical guidance.

**Table 2:** Serum IGF-I reference ranges and L, M, and S parameters of IGF-I in healthy female Chinese adults.

Age Group	L	M	S	Standard Deviation					Percentile	
				-2 SD	-1 SD	M	+1 SD	+2 SD	P_2.5_	P_97.5_
18	0.595	380.8	0.263	202.8	286.1	380.8	486.0	601.5	205.9	596.7
19	0.451	323.9	0.288	166.4	238.0	323.9	424.5	540.1	169.0	535.2
20–24	0.362	277.3	0.306	139.0	200.6	277.3	370.6	481.7	141.2	476.9
25–29	0.319	239.7	0.318	117.7	171.4	239.7	324.6	427.9	119.6	423.3
30–34	0.313	209.8	0.327	101.0	148.6	209.8	286.3	380.0	102.7	375.9
35–39	0.333	186.0	0.332	87.8	130.8	186.0	255.0	339.1	89.3	335.4
40–44	0.371	167.2	0.337	77.1	116.7	167.2	229.6	305.0	78.5	301.7
45–49	0.416	151.9	0.341	68.1	105.2	151.9	209.0	276.9	69.4	274.0
50–54	0.458	138.8	0.347	60.2	95.1	138.8	191.5	253.4	61.5	250.8
55–59	0.490	126.5	0.355	52.8	85.6	126.5	175.5	232.7	54.0	230.3
60–64	0.499	113.6	0.368	45.4	75.7	113.6	159.3	212.7	46.5	210.4
65–69	0.478	98.9	0.386	37.7	64.5	98.9	140.9	190.8	38.7	188.6
≥70	0.416	80.9	0.411	29.6	51.5	80.9	118.2	164.0	30.3	162.0

L, coefficient of skewness; M, Median; S, coefficient of variation. Both the standard deviation and percentile values are shown here for clinical guidance.

**Table 3:** Serum IGF-I reference ranges and L, M, and S parameters of IGF-I in all healthy Chinese adults.

Age Group	L	M	S	Standard Deviation					Percentile	
				-2 SD	-1 SD	M	+1 SD	+2 SD	P_2.5_	P_97.5_
18	0.634	374.1	0.245	208.1	286.7	374.1	469.7	573.1	211.1	568.8
19	0.533	314.8	0.282	160.8	231.9	314.8	409.5	515.7	163.5	511.3
20–24	0.455	267.4	0.307	130.1	192.1	267.4	356.5	459.6	132.3	455.2
25–29	0.399	230.2	0.322	109.3	163.1	230.2	311.6	408.3	111.2	404.1
30–34	0.362	201.6	0.329	95.1	142.0	201.6	275.1	363.8	96.7	360.0
35–39	0.341	180.1	0.331	85.0	126.7	180.1	246.4	327.0	86.5	323.5
40–44	0.336	164.0	0.330	77.8	115.6	164.0	224.2	297.6	79.1	294.4
45–49	0.342	151.8	0.328	72.1	107.1	151.8	207.2	274.4	73.4	271.5
50–54	0.359	141.8	0.329	67.0	100.0	141.8	193.5	255.8	68.2	253.1
55–59	0.385	132.5	0.333	61.4	92.8	132.5	181.3	239.9	62.5	237.4
60–64	0.416	122.3	0.345	54.3	84.3	122.3	168.8	224.2	55.3	221.8
65–69	0.451	109.6	0.365	45.2	73.5	109.6	153.7	206.0	46.2	203.8
≥70	0.487	92.7	0.397	34.0	59.6	92.7	133.3	181.5	34.8	179.4

L, coefficient of skewness; M, Median; S, coefficient of variation. Both the standard deviation and percentile values are shown here for clinical guidance.

**Fig 2:**
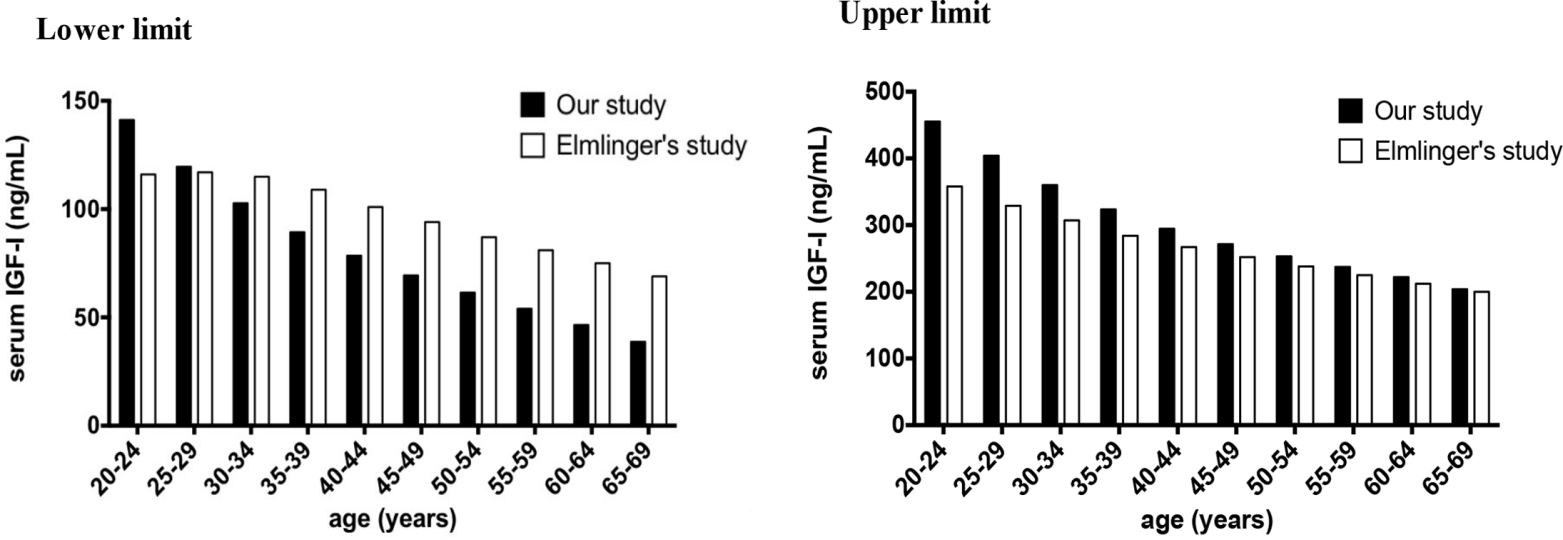
Comparison of the reference ranges for serum IGF-I levels between our study and Elmlinger’s study.

Next, we compared our present data with those obtained by Elmlinger in a study performed in a German population; these data were provided by the manufacturer and are used as the reference range in our daily clinical practice. As shown in Fig 2, significant differences were observed between our data and those obtained by Elmlinger in both the lower and upper limits (α<0.05 in Wilcoxon signed ranks test). Our lower limit (2.5^th^ percentiles) of IGF-1 was 25.2 ng/ml higher in the 20–24 age group than that in the study by Elmlinger, and the lower limit reached similar levels in the 25–29 age group. After the age of 30, the IGF-1 levels in our study were lower until the age of 69 years. The maximum difference was 30.3 ng/ml in the 65–69 age group. Regarding the upper limit (97.5^th^ percentiles), the IGF-1 levels in our study were significantly higher across all groups. The maximum difference was 70.5 ng/ml in the 20–24 age group. The IGF-I reference intervals (2.5^th^-97.5^th^ percentiles) in our study ranged from 102.5 ng/ml to 288.3 ng/ml across the 20–69 age groups, while these intervals were 47.0 ng/ml to 158.0 ng/ml in the study by Elmlinger. Our data had statistically similar medians with Elmlinger’s.

The reference ranges for the serum IGF-I levels in our present study were compared with those in the study by Elmlinger using Wilcoxon signed ranks test. Both the lower and upper limits were significantly different (α<0.05).

### Factors influencing the serum IGF-I levels

**Fig 3:**
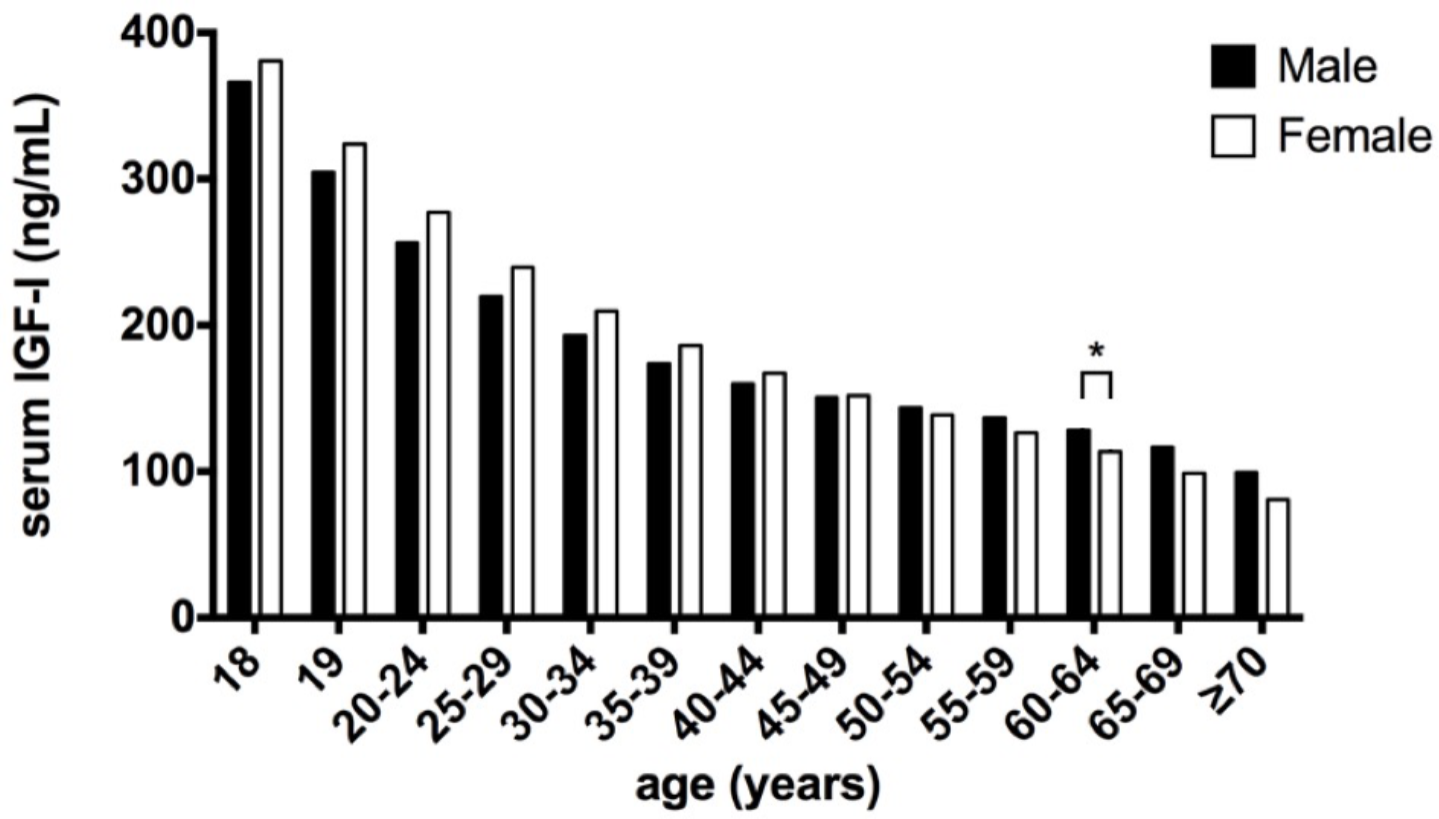
Comparison of the medians of the serum IGF-I levels between the males and females.

To compare the differences in the IGF-I levels between the sexes in each group, the SDS values of the serum IGF-I levels in each group were calculated using the LMS method and statistically analyzed using t-tests. As shown in Fig 3, the serum IGF-I levels in the males were lower than those in the females before the age of 45 years, reached similar levels as those in the females at the age of 45–49 years, were higher than those in the females after the age of 50 years and remained higher after the age of 70 years. This finding suggested that the rate of the decrease in the serum IGF-I level in the females was greater than that in the males.

The SDS serum IGF-I values in each group were calculated using the LMS method and compared by performing t-tests; the serum IGF-I levels in the males were significantly higher than those in the females only in the 60–64 age group (P = 0.0329).

However, a significant sex difference was observed in the serum IGF-I levels between the males and females only at the age of 60–64 years (P = 0.0329), and the upper limit (+2 SD) and lower limit (-2 SD) in the males was 20.7 ng/ml and 19.1 ng/ml higher than that in the females, respectively.

Finally, a multiple linear regression model was established to observe the effects of age, sex, BMI, geographical region, etc. on the serum IGF-1 levels. As shown in Table 4, the adjusted R^2^ of the model was 0.416, and the standard error of the estimate was 62.6. The interacting item of BMI-age (multiply BMI by age) was introduced to eliminate the interaction effects between BMI and age. Consistent with the abovementioned results, the serum IGF-I levels were negatively correlated with age (P<0.001); the levels decreased by 4.5 ng/ml each year after adjusting for sex, geographical region and BMI. A regional distinction was also observed in this model, as evidenced by serum IGF-I levels in Guizhou that were 7.0 ng/ml higher than those in Beijing (P<0.01). With respect to BMI, all subjects were divided into quintiles (18.51–19.20 kg/m^2^, 19.21–20.96 kg/m^2^, 20.97–22.80 kg/m^2^, 22.81–24.71 kg/m^2^ and 24.72–27.99 kg/m^2^), and the relationship between IGF-I level and BMI category was observed. The results showed that the IGF-I levels were significantly inversely associated with the BMI category. Subjects in a higher BMI category had lower serum IGF-I levels than those in a lower BMI category, and the average intra-group decrease was 16.6 ng/ml (P<0.001). Further analysis showed that the decrease in the serum IGF-I levels with age was 0.4 ng/ml lower in the subjects with a higher BMI when BMI and age were set as interacting variables (P<0.001). Finally, no significant differences were observed in the IGF-1 levels between the sexes, although the decrease in the females was steeper than that in the males (P = 0.855).

**Table 4:** Multiple linear regression model of serum IGF-I and its influencing factors.

Model	Unstandardized Coefficients		t	Sig.	VIF
	β	Stand. Error			
(Constant)	378.7	8.8	43.3	<0.001	
Age	-4.5	0.2	-24.1	<0.001	5.5
Sex (0/1)	-0.5	2.5	-0.2	0.855	1.1
Region (0/1)	7.0	2.8	2.5	0.012	1.3
BMI (1/2/3/4/5)	-16.6	2.8	-6.0	<0.001	11.1
BMI (1/2/3/4/5)-Age	0.4	0.1	6.0	<0.001	18.2

R^2^ = 0.418, adjusted R^2^ = 0.416, standard error of the estimate = 62.6. ANOVA analysis: F = 399.3, Significance P<0.001. VIF, Variance Inflation Factor. Sex: 0-male and 1-female. Region: 0-Beijing and 1-Guizhou. All subjects were divided into five BMI groups (1, 18.51–19.21 kg/m^2^; 2,19.21–20.96 kg/m^2^; 3, 20.97–22.80 kg/m^2^; 4, 22.81–24.71 kg/m^2^; and 5, 24.72–27.99 kg/m^2^) using quintile division.

## Discussion

Accurate measurements of serum IGF-I levels and established reliable normative reference intervals are crucial for the diagnosis and treatment of disorders, including acromegaly (GH excess) and GHD (GH deficiency). However, normal reference ranges for serum IGF-I based on a large sample of the Chinese population are scarce. Therefore, in our present study, 2791 male and female Chinese subjects aged from 18 to over 70 years from North and South China were recruited, and the serum IGF-I levels were measured using a chemiluminescent assay. Subsequently, the serum IGF-I reference ranges and L, M, and S parameters of IGF-I were obtained in both sexes using the LMS method; the values of the 2.5^th^ and 97.5^th^ percentiles are also listed for clinical guidance. To the best of our knowledge, this was the first study to establish age- and sex-specific reference ranges for serum IGF-I on a large scale in a Chinese population using a chemiluminescent assay, and the sample size was also the largest in an Asian population.

It has been well documented that different commercial IGF-I assay kits could provide different results for the same samples with up to a 2.5-fold difference between the lowest and highest values [12]. Radioimmunoassays, immunoradiometric assays and enzyme linked immunosorbent assays (ELISAs) for IGF-I measurements have been established. Chanson et al. recently measured the serum IGF-I levels in healthy French adults using six immunoassays and found that the reference intervals of the six commercial IGF-1 assay kits were markedly different, even though they were obtained from the same healthy population [13]. The Nichols Advantage (chemiluminescence assay) was once thought to be the ‘gold standard’ for IGF-I measurement but soon exited the markets. In 2008, Krebs et al. compared certain popular techniques available on the market and found that the IMMULITE and IDS assays had the greatest consistency with the Nichols Advantage [14]. In Chanson’s study, the concordances between IMMULITE and other methods were good (0.50 to 0.64), and the percentages of observed agreement were quite high (94.83% to 96.08%) [13]. Therefore, we selected IMMULITE 2000 for our study because this method is fast, highly automated, and had been widely used in both our country and globally. In addition, intra- and inter-assay controls developed in our laboratory were used in the present measurements to assess the accuracy of each assay. Consequently, a 3.04% CV confirmed the accuracy of our assays.

The previous reference ranges for serum IGF-I provided by the IMMULITE 2000 manufacturer were obtained from a study conducted by Elmlinger in Germany in 2004 [15]. In our present study, we compared our data with those obtained by Elmlinger and found that there was a significant difference in both the lower and upper limits. The differences between the two studies could be explained as follows: the difference in the genetic background between Chinese and German populations, our stricter inclusion criteria that limited BMI, and our superior statistical methods. Because the upper and lower limits of serum IGF-I are used to diagnose and treat acromegaly and adult GHD, respectively, our new reference ranges could change the present clinical practices. For acromegaly, the threshold for diagnosis and the targeted value for treatment could be elevated. These values are recommended to be set at +2 SD, which is higher in our new reference ranges [16]. For adult GHD, the threshold for diagnosis could be elevated for subjects aged 20–24 and reduced for subjects above the age of 30 years.

In the present study, we demonstrated that the serum IGF-I levels in adults gradually decrease with age in both males and females. The serum IGF-I levels at the age of 45–49 years were approximately half of those at the age of 18 years and approximately one-fourth of those at the age of 18 years in subjects older than 70 years. The decrease in IGF-1 was steeper in the females, who initially had higher levels than the males but had lower levels than the males after the age of 50 years. However, the serum IGF-I levels in the males were significantly higher than those in the females only in the 60-64-year-old age group. Consistent with our observation, studies performed by Chanson et al. in a French population also demonstrated a continuous decline in circulating IGF-I levels throughout adulthood, which was accelerated in females compared with that in males [13]. Similar results were obtained in several studies performed in Caucasians [6] and Brazilians [17,18].

Sex differences were observed in the serum IGF-1 levels in previous studies [6,19,20]. It was hypothesized that the decrease in estrogen levels with age in females might explain the sex differences [20] because estrogen is well known to play an important role in regulating the GH/IGF-I axis in both sexes [21]. However, we observed only a trend in the sex difference in the serum IGF-1 levels, and no significant differences were obtained in most age groups, except for the 60- to 64-year-old group. A possible explanation for the discrepancy between our work and previous reports might be that we did not exclude females taking oral contraceptives, which might result in a decrease in the serum IGF-I levels because contraceptive pills contain ethinylestradiol, which could down-regulate serum IGF-I by 70% in pre-menopausal women [22]. Nevertheless, this influence is likely limited because the incidence of women taking oral contraceptives was only 0.98% in 2010 in China [23], which is considerably less than the 17.3–39.4% observed in developed countries and 5.9% observed in other developing countries [19,24]. The use of oral contraceptives might explain why we failed to observe a significant difference.

BMI reflects both the body composition and nutritional status. Serum GH has been previously reported to be decreased and serum IGF-I has been reported to be increased in obese individuals [25]. Fasting [26] or weight gain [27,28] could decrease or increase human serum IGF-I, respectively. However, the correlation between serum IGF-I and BMI remains inconsistent across epidemiological studies. Several studies have reported an inverse relationship [8,29–32]. Landin-Wllhelmsen et al. [29] was the first to report this relationship in 1994 after studying 392 healthy adults aged 25–34 in Sweden. The largest study thus far was conducted in 2009 by Faupel-Badger et al. [30]. These authors recruited 5803 American adults, including non-Hispanic whites, non-Hispanic blacks and Mexican-Americans. An inverse association was observed across all race/ethnicity and sex subgroups [30]. Consistent with these studies, our study also found that the IGF-1 levels were negatively associated with BMI. Subjects with a higher BMI could have much lower IGF-1 levels. To the best of our knowledge, this was the first study to report an inverse association between serum IGF-I and BMI in an Asian population.

However, several studies support the presence of a positive [31], non-linear [32–35] or null [36–38] relationship between serum IGF-I levels and BMI. The largest study was conducted by Crowe et al. in 2011 [33]. These authors enrolled 1142 males and 3589 females from Europe, and the serum IGF-I levels were observed to be strongly correlated with BMI. The peak value of serum IGF-I appeared at 26–27 kg/m^2^ [33]. The most likely explanation for this discrepancy might be the different cut-off points used to define the BMI categories, the different genetic backgrounds, and the inclusion and exclusion criteria.

Notably, most studies investigating the relationship between serum IGF-I and BMI were conducted in Western countries, where BMIs are usually over 24 kg/m^2^. Limited data regarding Asians have been reported. Probst-Hensch et al. [39] studied 638 healthy subjects aged 45 to 75 in Singapore. The average BMI was 22.5 kg/m^2^ for males and 22.9 kg/m^2^ for females. Teramukai et al. [40] studied 616 Japanese males between 45 and 55 years of age, and the BMI ranged from 16.42 to 32.83 kg/m^2^. Our study provides valuable data regarding healthy subjects (mean BMI was 23.3±2.4 kg/m^2^ for males and 22.6±2.4 kg/m^2^ for females) with a broader age spectrum (18 to 87 years old). Notably, our study restricted the BMI (18.5–28 kg/m^2^). Our data may be more convincing if subjects with extreme BMIs (anorexia or adiposity) were included.

Our study also found a geographical regional distinction in the serum IGF-I level, which has rarely been discussed in other studies. Bayram et al. [41] studied Caucasians in seven areas in Turkey and found significant regional differences in both males and females. The impact of region on serum IGF-I might be due to racial differences. In a 20-year cohort study, Borofsky et al. [42] showed that genetics strongly influenced the levels of serum IGF-I. By measuring serum IGF-I levels in 248 twins from Sweden, Hong et al. [43] found that genetic factors could influence serum IGF-I to a degree of 64%, which is greater than that achieved by other endocrine markers, such as insulin (48%) or IGFBP1 (36%). However, other factors, such as diet, body composition or the use of oral contraceptives, might also be responsible for the variance in the IGF-1 levels. In the study by Bayram et al. mentioned above [41], all subjects were Caucasian. However, subjects in certain sectors had a high protein diet, while the diets of those in other areas were mainly composed of vegetarians. In our study, subjects in Beijing and Guizhou were mainly from the North and South China Han population, respectively. Genetic differences might explain the regional distinction because genome-wide association studies have shown that there was a “north-south” population structure and a close correlation between geography and the genetic structure of the Han Chinese population [44]. Furthermore, people in North China prefer consuming meat and mutton, whereas people in the south are more likely to be vegetarians. More information regarding dietary preferences should be obtained in future studies.

The reference values established in our study were constructed using the LMS method, which is one of the most widely applied approaches for age-related reference intervals. The LMS method was first proposed in 1988 by Cole. The parameters were estimated separately within each age group by maximum likelihood and then smoothed across age. This approach was adopted in our study for the following advantages. First, the distribution of serum IGF-I in the population was skewed, and the LMS method can produce convincing centile curves with skewed data. Second, the LMS method can draw any centile curve to perfectly meet the clinical needs. Third, the LMS method can calculate the SDS according to the measured values. Finally, the distribution of the curves constructed using the LMS method was dependent on the overall parameters, which could reduce the influence of grouping. The goodness of fit test is shown in S4 Table. This test showed that the age-specific difference between the simulated centiles and the measured centiles was very limited, and most (34 of 39) differences were less than 10%.

Our study had several limitations. First, the assays were calibrated using the old standard IRP 87/518 rather than 02/254 as recommended since 2011 [45]. Theoretically, the use of the new recombinant calibrator should be associated with lower absolute concentrations reported. Because data regarding the Chinese population are scarce, efforts should be paid to establishing reference ranges for IGF-I levels using the current standard. Second, the Siemens Immulite IGF-I assay had encountered dramatic lot-to-lot variability, making the use of previously published reference intervals less reliable [46]. This issue is particularly prevalent to our comparison of our data and those obtained by Elmlinger, which were published more than 15 years ago. This between-lot variability might make the monitoring of IGF-I less effective.

In conclusion, our study established age- and sex-specific reference ranges for serum IGF-I in a Chinese population and found that age, sex, BMI and geographical region all had a notable influence on the circulating IGF-1 levels. These results provide a foundation for the diagnosis and treatment of several diseases, including acromegaly and adult growth hormone deficiency in Asia.

## Supporting information

S1 Fig Measurements of inter-and intra-assay controls made by ourselves. The intra-and inter-assay controls were made by ourselves. Serum with unknown IGF-I concentration were pooled together, then mixed thoroughly and dispensed into 47 Eppendorf tubes. When the measurements were conducted, these controls were evenly inserted into the sample sequence and were measured. The mean value was 216.7 ng/ml and the coefficient of variation (CV) was 3.0%.
(DOCX)

S1 Table Distribution of subjects of both genders. Subjects of both genders were divided into 13 groups according to the age (18, 19, 20–24, 25–29, 30–34, 35–39, 40–44, 45–49, 50–54, 55–59, 60–64, 65–69 and ≥70). Each group contained more than 30 subjects except for the age group 19 (9 males, 15 females, 24 in total).
(DOCX)

S2 Table Measurement of inter-assay controls derived from the manufacturer. CV = coefficient of variation. Target values of low, middle and high concentration (mean ± SD) were 62.3 ±7.61 ng/ml, 270 ±26.7 ng/ml and 575 ±47.2 ng/ml, respectively. All the values were within ±2 SD values defined by the manufacturer.
(DOCX)

S3 Table Baseline information of the subjects (mean±SD). TC = total cholesterol, TG = triglyceride, HDL = high-density lipoprotein, LDL = low-density lipoprotein, Fat% = body fat percentage. *P<0.01 *vs* females.
(DOCX)

S4 Table Comparison of simulated centiles and measured centiles of IGF-I. Difference = (Simulated centile–Measured centile)/Measured centile.
(DOCX)
