# Supplementary material for: Reference ranges for serum insulin-like growth factor I (IGF-I) in healthy Chinese adults
Source: PLoS One. 2017 Oct 4;12(10):e0185561. doi: 10.1371/journal.pone.0185561 (PMC5627923; doi:10.1371/journal.pone.0185561)
Supplement: S1 Fig — The intra-and inter-assay controls were made by ourselves. Serum with unknown IGF-I concentration were pooled together, then mixed thoroughly and dispensed into 47 Eppendorf tubes. When the measurements were conducted, these controls were evenly inserted into the sample sequence and were measured. The mean value was 216.7 ng/ml and the coefficient of variation (CV) was 3.0%. (DOCX) [file pone.0185561.s001.docx]

**Supplementary Materials**

**Figure S1. Measurements of inter-and intra-assay controls made by ourselves**

The intra-and inter-assay controls were made by ourselves. Serum with unknown IGF-I concentration were pooled together, then mixed thoroughly and dispensed into 47 Eppendorf tubes. When the measurements were conducted, these controls were evenly inserted into the sample sequence and were measured. The mean value was 216.7 ng/ml and the coefficient of variation (CV) was 3.0 %.
