# Supplementary material for: Reference ranges for serum insulin-like growth factor I (IGF-I) in healthy Chinese adults
Source: PLoS One. 2017 Oct 4;12(10):e0185561. doi: 10.1371/journal.pone.0185561 (PMC5627923; doi:10.1371/journal.pone.0185561)
Supplement: S1 Table — Subjects of both genders were divided into 13 groups according to the age (18, 19, 20–24, 25–29, 30–34, 35–39, 40–44, 45–49, 50–54, 55–59, 60–64, 65–69 and ≥70). Each group contained more than 30 subjects except for the age group 19 (9 males, 15 females, 24 in total). (DOCX) [file pone.0185561.s002.docx]

**Supplementary Materials**

**Table S1. Distribution of subjects of both genders**

| Age group | 18 | 19 | 20-24 | 25-29 | 30-34 | 35-39 | 40-44 |
| --- | --- | --- | --- | --- | --- | --- | --- |
| Male | 30 | 9 | 54 | 96 | 126 | 149 | 160 |
| Female | 51 | 15 | 84 | 129 | 128 | 203 | 175 |
| Total | 81 | 24 | 138 | 225 | 254 | 352 | 335 |
|  | | | | | | | |
| Age group | **45-49** | **50-54** | **55-59** | **60-64** | **65-69** | **≥70** | **Total** |
| Male | 172 | 133 | 157 | 92 | 76 | 85 | 1339 |
| Female | 173 | 134 | 120 | 105 | 59 | 76 | 1452 |
| Total | 345 | 267 | 277 | 197 | 135 | 161 |  |

Subjects of both genders were divided into 13 groups according to the age (18, 19, 20-24, 25-29, 30-34, 35-39, 40-44, 45-49, 50-54, 55-59, 60-64, 65-69 and ≥70). Each group contained more than 30 subjects except for the age group 19 (9 males, 15 females, 24 in total).
