# Supplementary material for: Reference ranges for serum insulin-like growth factor I (IGF-I) in healthy Chinese adults
Source: PLoS One. 2017 Oct 4;12(10):e0185561. doi: 10.1371/journal.pone.0185561 (PMC5627923; doi:10.1371/journal.pone.0185561)
Supplement: S2 Table — CV = coefficient of variation. Target values of low, middle and high concentration (mean ± SD) were 62.3 ±7.61 ng/ml, 270 ±26.7 ng/ml and 575 ±47.2 ng/ml, respectively. All the values were within ±2 SD values defined by the manufacturer. (DOCX) [file pone.0185561.s003.docx]

**Supplementary Materials**

**Table S2. Measurement of inter-assay controls derived from the manufacturer**

|  | | **Concentration (ng/ml)** | | |
| --- | --- | --- | --- | --- |
|  |  | **Low** | **Middle** | **High** |
| **Date** | 04/12/2016 | 56.8 | 246 | 534 |
|  | 04/13/2016 | 54.9 | 248 | 536 |
|  | 04/14/2016 | 55.2 | 254 | 537 |
|  | 04/15/2016 | 55.5 | 256 | 534 |
|  | 04/16/2016 | 60.4 | 269 | 520 |
|  | 04/17/2016 | 60.2 | 252 | 646 |
|  | 04/21/2016 | 56.1 | 250 | 526 |
|  | 04/22/2016 | 55.5 | 257 | 543 |
|  | 04/23/2016 | 59.4 | 287 | 533 |
|  | 04/26/2016 | 64.7 | 246 | 550 |
|  | 05/06/2016 | 57.4 | 269 | 576 |
|  | 05/07/2016 | 56.2 | 244 | 603 |
| **Mean ± SD** | | 57.7 ± 2.94 | 256.5 ± 12.65 | 553.2 ± 37.26 |
| **CV%** | | 5.09 | 4.93 | 6.73 |

CV = coefficient of variation. Target values of low, middle and high concentration (mean ± SD) were 62.3 ±7.61 ng/ml, 270 ±26.7 ng/ml and 575 ±47.2 ng/ml, respectively. All the values were within ±2 SD values defined by the manufacturer.
