# Supplementary material for: Reference ranges for serum insulin-like growth factor I (IGF-I) in healthy Chinese adults
Source: PLoS One. 2017 Oct 4;12(10):e0185561. doi: 10.1371/journal.pone.0185561 (PMC5627923; doi:10.1371/journal.pone.0185561)
Supplement: S3 Table — TC = total cholesterol, TG = triglyceride, HDL = high-density lipoprotein, LDL = low-density lipoprotein, Fat% = body fat percentage. *P<0.01 vs females. (DOCX) [file pone.0185561.s004.docx]

**Supplementary Materials**

**Table S3. Baseline information of the subjects（mean±SD）**

|  | Male (n=1339) | Female (n=1452) | Total (n=2791) |
| --- | --- | --- | --- |
| age | 46.2±14.7* | 43.7±14.7 | 44.9±14.8 |
| BMI（kg/m^2^） | 23.3±2.4* | 22.6±14.7 | 22.9±2.5 |
| height（m） | 170.0±16.5 | 155.9±6.5 | 162.7±14.3 |
| TC（mmol/l） | 4.83±0.98* | 4.49±1.27 | 4.68±1.13 |
| TG（mmol/l） | 1.7±2.9* | 1.28±1.9 | 1.52±2.52 |
| HDL（mmol/l） | 1.25±0.35 | 1.36±0.8^#^ | 1.3±0.59 |
| LDL（mmol/l） | 3.32±9.7* | 2.65±1.04 | 3.03±7.35 |
| Fat（%） | 22.22±11.34 | 27.04±11.63^#^ | 24.18±11.7 |

TC = total cholesterol, TG = triglyceride, HDL = high-density lipoprotein, LDL = low-density lipoprotein, Fat% = body fat percentage. *P<0.01 *vs* females.
