# Supplementary material for: Reference ranges for serum insulin-like growth factor I (IGF-I) in healthy Chinese adults
Source: PLoS One. 2017 Oct 4;12(10):e0185561. doi: 10.1371/journal.pone.0185561 (PMC5627923; doi:10.1371/journal.pone.0185561)
Supplement: S4 Table — Difference = (Simulated centile–Measured centile)/Measured centile. (DOCX) [file pone.0185561.s005.docx]

**Supplementary Materials**

**Table S4. Comparison of simulated centiles and measured centiles of IGF-I**

| Age group | P_2.5_ | | | P_50_ | | | P_97.5_ | | |
| --- | --- | --- | --- | --- | --- | --- | --- | --- | --- |
|  | Measured centile | Simulated centile | difference | Measured centile | Simulated centile | difference | Measured centile | Simulated centile | difference |
| 18 | 208.0 | 211.1 | 0.01 | 367.0 | 374.1 | 0.02 | 566.0 | 568.8 | 0.00 |
| 19 | 198.6 | 163.5 | -0.18 | 334.0 | 314.8 | -0.06 | 541.3 | 511.3 | -0.06 |
| 20-24 | 111.4 | 132.3 | 0.19 | 256.0 | 267.4 | 0.04 | 487.4 | 455.2 | -0.07 |
| 25-29 | 108.2 | 111.2 | 0.03 | 224.0 | 230.2 | 0.03 | 395.0 | 404.1 | 0.02 |
| 30-34 | 88.6 | 96.7 | 0.09 | 205.0 | 201.6 | -0.02 | 373.1 | 360 | -0.04 |
| 35-39 | 101.0 | 86.5 | -0.14 | 184.0 | 180.1 | -0.02 | 316.9 | 323.5 | 0.02 |
| 40-44 | 78.5 | 79.1 | 0.01 | 162.0 | 164 | 0.01 | 282.7 | 294.4 | 0.04 |
| 45-49 | 72.4 | 73.4 | 0.01 | 151.0 | 151.8 | 0.01 | 260.2 | 271.5 | 0.04 |
| 50-54 | 62.5 | 68.2 | 0.09 | 142.0 | 141.8 | 0.00 | 259.8 | 253.1 | -0.03 |
| 55-59 | 54.2 | 62.5 | 0.15 | 137.0 | 132.5 | -0.03 | 255.0 | 237.4 | -0.07 |
| 60-64 | 61.1 | 55.3 | -0.09 | 117.0 | 122.3 | 0.05 | 215.4 | 221.8 | 0.03 |
| 65-69 | 47.9 | 46.2 | -0.04 | 112.0 | 109.6 | -0.02 | 218.7 | 203.8 | -0.07 |
| ≥70 | 27.8 | 34.8 | 0.25 | 92.6 | 92.7 | 0.00 | 184.0 | 179.4 | -0.03 |

Difference = (Simulated centile – Measured centile)/Measured centile.
